# Exploring 2.5-Year Trajectories of Functional Decline in Older Adults by Applying a Growth Mixture Model and Frequency of Outings as a Predictor: A 2010–2013 JAGES Longitudinal Study

**DOI:** 10.2188/jea.JE20170230

**Published:** 2019-02-05

**Authors:** Junko Saito, Naoki Kondo, Masashige Saito, Daisuke Takagi, Yukako Tani, Maho Haseda, Takahiro Tabuchi, Katsunori Kondo

**Affiliations:** 1 Department of Health and Social Behavior, Department of Health Education and Health Sociology, School of Public Health, The University of Tokyo, Tokyo, Japan; 2 Faculty of Social Welfare, Nihon Fukushi University, Aichi, Japan; 3 Department of Global Health Promotion, Tokyo Medical and Dental University, Tokyo, Japan; 4 Research Fellow of Japan Society for the Promotion of Science, Tokyo, Japan; 5 Cancer Control Center, Osaka International Cancer Institute, Osaka, Japan; 6 Department of Social Preventive Medical Sciences, Center for Preventive Medical Sciences, Chiba University, Chiba, Japan; 7 Center for Well-being and Society, Nihon Fukushi University, Aichi, Japan; 8 Department of Gerontological Evaluation, Center for Gerontology and Social Science, National Center for Geriatrics and Gerontology, Aichi, Japan

**Keywords:** functional decline, trajectory, homebound persons, older people, Japan

## Abstract

**Background:**

We explored the distinct trajectories of functional decline among older adults in Japan, and evaluated whether the frequency of outings, an important indicator of social activity, predicts the identified trajectories.

**Methods:**

We analyzed data on 2,364 adults aged 65 years or older from the Japan Aichi Gerontological Evaluation Study. Participants were initially independent and later developed functional disability during a 31-month follow-up period. We used the level of long-term care needs certified in the public health insurance system as a proxy of functional ability and linked the fully tracked data of changes in the care levels to the baseline data. A low frequency of outings was defined as leaving one’s home less than once per week at baseline. We applied a growth mixture model to identify trajectories in functional decline by sex and then examined the association between the frequency of outings and the identified trajectories using multinomial logistic regression analysis.

**Results:**

Three distinct trajectories were identified: “slowly declining” (64.3% of men and 79.7% of women), “persistently disabled” (4.5% and 3.7%, respectively), and “rapidly declining” (31.3% and 16.6%, respectively). Men with fewer outings had 2.14 times greater odds (95% confidence interval, 1.03–4.41) of being persistently disabled. The association between outing frequency and functional decline trajectory was less clear statistically among women.

**Conclusions:**

While the majority of older adults showed a slow functional decline, some showed persistent moderate disability. Providing more opportunities to go out or assistance in that regard may be important for preventing persistent disability, and such needs might be greater among men.

## INTRODUCTION

Population aging has been observed and is accelerating worldwide. The World Health Organization has proposed global strategies to maintain the functional ability and intrinsic capacity of older adults by removing barriers to participation and compensating for capacity losses.^1^ Japan is the global leader in the rapidity of population aging: in 2015, the proportion of older people aged 65 or more was 26.7%, and by 2060, this proportion is expected to reach upwards of 40%.^2^ The Japanese government implemented a public long-term care insurance (LTCI) system in 2000 and has since faced the challenges of both sustaining the system financially and improving its performance in maintaining and improving the functional ability of those insured.

To overcome these challenges, it is necessary to determine how older people’s functional ability changes and what environments are most conducive to declines. Han et al identified five distinct trajectories of functional ability among community-dwelling older adults: independent, consistently low or high disability, and a gradual change towards low or high disability.^3^ In Japan, Liang et al classified three trajectories of functional impairment over 10 years: minimal function decrement, early onset and accelerated in the 80s, and late onset in the 70s–80s.^4^ However, comparatively few studies have identified these trajectories using an objective indicator of older adults’ functional ability.

Potential predictors of the trajectories in older adults’ functional ability are biological, socioeconomic, and psychosocial in nature.^3^^–^^5^ Among these factors, the psychosocial risk factors (eg, social relationships) are especially important to consider when planning interventions in community settings. A previous systematic review suggested that having few social contacts is a critical risk factor of functional disability in older adults.^6^ However, we still have relatively little understanding of the association between social connections and trajectories in functional disability in older adults.^7^

To improve our understanding of this association, we focused on the frequency of outings as a proxy indicator of the level of social activity and connectedness. Research has shown that fewer outings predicts the incidence of functional disability, cognitive decline, and premature mortality among older adults.^8^ In Japan, there is a condition called “tojikomori” where individuals do not go out frequently, despite not having functional problems in the physical, mental, or cognitive domains.^9^ Tojikomori is considered preventable because it is related to individuals’ social relationships and environment.^10^ In this study, we explored the distinct trajectories of functional decline—measured using an objective evaluation scheme—and whether going out less often in the absence of functional problems predicts these identified trajectories.

## METHODS

### Study subjects

**Figure 1.**
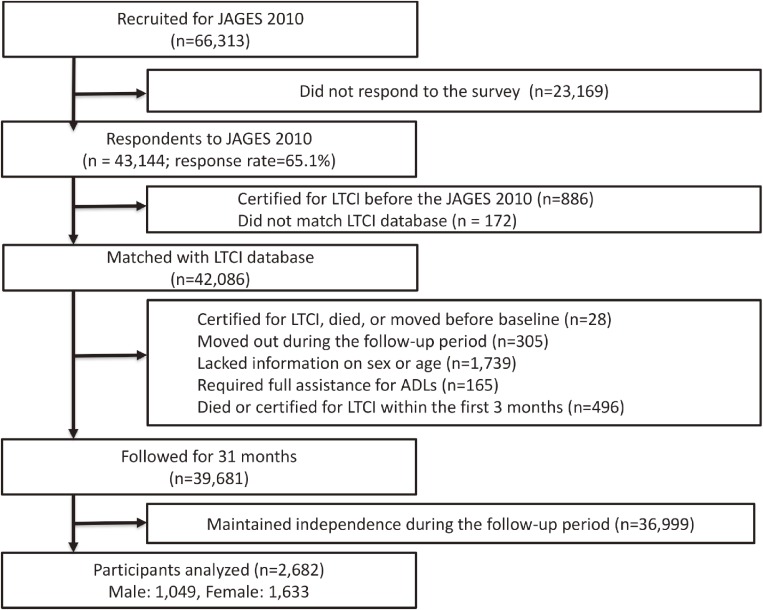
Flow chart of participants in this study

We used data from the Japan Gerontological Evaluation Study (JAGES), which is an on-going longitudinal study targeting community-dwelling and functionally independent older adults aged ≥65 years (see the flow chart of participation in Figure 1). Functional independency was defined as not being certified for Japan’s LTCI system. The JAGES survey conducted in 2010 served as the baseline for our longitudinal observations, with which we combined individual LTCI data provided from 12 municipalities in six prefectures. Within these 12 municipalities, 26,690 older adults were randomly selected across six municipalities, and a census was taken in the other six municipalities (yielding a further 39,623 older adults). Of the 66,313 adults surveyed, 43,144 returned the questionnaire (response rate 65.1%); of these, the data of 42,086 were matched with the LTCI database. We excluded those who had been certified for LTCI or had died or moved before the survey date (*n* = 28), who moved out from their current residence during the follow-up period (*n* = 305), who lacked information on sex or age (*n* = 1,739), who required full assistance for activities of daily living (ADLs) (*n* = 165), or who died or were certified as needing long-term care within the first 3 months from baseline (*n* = 496). Finally, we excluded those who maintained their independence during the follow-up period (*n* = 36,999). This was because the proportion was too large (87.9% of those who had data matched with the LTCI database over 31 months) to ensure accurate classification of the trajectories of individuals requiring long-term care. Thus, a total of 2,682 participants were analyzed. The JAGES protocol was reviewed and approved by the ethics committee on research of human subjects at Nihon Fukushi University (No. 10-05), and the use of the JAGES data for this study was approved by the Ethics Committee of the Faculty of Medicine, University of Tokyo (No. 10555).

### Assessment of functional ability

We used the certified long-term care levels of the national LTCI as an index of functional ability. According to this system, individuals are classified into one of seven care levels based on the severity of their physical and cognitive disability via a home-visit interview and physician examination. A lower care level implied worse functional ability. Moreover, the seven care levels are framed by independence (level 8) and death (level 0), such that functional ability falls on a continuum between these two states. For instance, level 3 (youkaigo 3) refers to “complete support needed in toileting, bathing, dressing, and all other basic ADLs.” The details of each care level are provided elsewhere.^11^

The validity period of each care level is, in principle, 6 months for the initial certification and 12 months for each subsequent certification. However, older adults can apply for a re-evaluation of their care level whenever they wish. We obtained information on the date of certification and the care levels until 31 months after baseline or death from the LTCI database. Then, we aggregated the information on care levels for every 3 months to create equidistant intervals of observation for the trajectory analysis.

### Low frequency of outings

We defined a low frequency of outings as leaving one’s home less than once per week.^12^^,^^13^ The frequency of leaving home was measured at the time of baseline survey using a single question: “How often do you usually go outside the house?”. Possible answers were “almost every day,” “2 to 3 times per week,” “about once per week,” “1 to 2 times per month,” “several times in a year,” and “never”. Although this single-question measure has not been formally validated, it has been widely used in previous studies,^8^^,^^14^ as well as in the process of certification by the government for long-term nursing care.^15^

### Covariates

The potential confounding factors were socio-demographic characteristics (ie, age, marital status, education years, equivalent household income, household composition, and urbanization [population density]), health status (self-reported body mass index [BMI], current medical history [at least one of the following diseases: stroke, cancer, bone or heart diseases], self-rated health, and depressive symptoms according to a score of 6 or more on the short version of the Geriatric Depression Scale [GDS-15]),^16^^,^^17^ and physiological variables (intellectual activity and instrumental ADLs assessed using the Tokyo Metropolitan Institute of Gerontology Index of Competence).^18^

### Modeling and analysis

First, we used a growth mixture model (GMM) to identify the trajectories in functional ability by sex. GMM is considered more appropriate than is a conventional latent growth curve model for the investigation of individual trajectories when those trajectories are heterogeneous. This is because it assumes that the population consists of distinct sub-groups of trajectories.^19^ A multivariate skewness/kurtosis test (SK test) revealed that the distribution of long-term care levels was not normal, so we applied a non-normal GMM, which can fit non-normal data considerably better than can normal mixture models.^20^

The time in months from baseline until the date of certification of long-term care levels was used as the time scale; this ranged from 4 to 31 months (ie, 10 time points). We constructed the trajectory models according to the number of trajectory classes (2 to 4) and trajectory shape (linear, quadratic, or cubic) and chose the best-fitting model according to the lowest Bayesian information criterion (BIC), adjusted Lo-Mendell-Rubin likelihood ratio test (Adj. LMR-LRT), and study objective (identification of comparable trajectories of functional ability). Then, we evaluated the average posterior probabilities of each class (a value of above 0.70 indicated good discrimination). Age was included as a covariate in the GMM. The GMM analysis was conducted using Mplus 7 (Muthén & Muthén, Los Angeles, CA, USA).

We then used multinomial logistic regression analysis, stratified by sex, to calculate the odds ratios (ORs) and 95% confidence intervals (CIs) for exhibiting a pattern of deterioration (ie, the base outcome was “slowly declining”). In model 1, we adjusted for the socio-demographic characteristics, and in model 2, we added health status and the physiological variables to model 1. We then performed a sensitivity analysis, which involved performing the logistic regression analysis again but excluding participants aged 85 years or older, for whom less frequent outings and deterioration in functional ability are considered natural phenomena associated with aging. We also excluded participants who died in the first 6 months from baseline because rapid deterioration of function and death within 6 months is likely the result of disease. Finally, we controlled for long-term care level at the first certification. Because the baseline care levels differed substantially between “slow decline toward low disability” and “persistently moderate decline,” we wanted to check if the association between outing frequency and trajectories in functional disability remained even after controlling for the initial long-term care levels. Two-sided alphas of 0.05 were considered statistically significant. The regression analyses were conducted using STATA 14.1 (Stata Corp, College Stations, TX, USA).

## RESULTS

The baseline characteristics of the 2,682 participants (1,049 men and 1,633 women) are shown in Table 1. Men and women had average ages of 79.0 and 80.2 years, respectively. Furthermore, 16.6% of men and 19.4% of women had a low frequency of outings. During the 31-month follow-up period, 17.6% of men and 14.1% of women were certified as completely dependent for many ADLs (ie, had care level 3 or lower [youkaigo 3 to 5]), and 27.3% of men and 10.5% of women died.

**Table 1.** Characteristics of study participants by trajectory patterns and sex at baseline

Characteristics of study participants												

	Men (*n* = 1,049)						Women (*n* = 1,633)					
	
	Slowly declining (*n* = 674)		Persistently disabled (*n* = 47)		Rapidly declining (*n* = 328)		Slowly declining (*n* = 1,301)		Persistently disabled (*n* = 61)		Rapidly declining (*n* = 271)	
					
	*n*	%	*n*	%	*n*	%	*n*	%	*n*	%	*n*	%
Age												
65–74	154	22.8	8	17.0	92	28.0	213	16.4	11	18.0	60	22.1
75–84	389	57.7	31	66.0	156	47.6	760	58.4	34	55.7	150	55.4
85–	131	19.4	8	17.0	80	24.4	328	25.2	16	26.2	61	22.5
Marital status												
Married	490	72.7	40	85.1	236	72.0	469	36.0	28	45.9	101	37.3
Single	120	17.8	5	10.6	60	18.3	693	53.3	27	44.3	145	53.5
Missing	64	9.5	2	4.3	32	9.8	139	10.7	6	9.8	25	9.2
Education (years)												
≤9	346	51.3	33	70.2	177	54.0	718	55.2	38	62.3	165	60.9
≥10	267	39.6	10	21.3	114	34.8	417	32.1	14	23.0	73	26.9
Missing/others	61	9.1	4	8.5	37	11.3	166	12.8	9	14.8	33	12.2
Equivalized income												
1.99 million yen or less	292	43.3	26	55.3	145	44.2	480	36.9	24	39.3	103	38.0
2 million yen or more	213	31.6	16	34.0	98	29.9	315	24.2	14	23.0	70	25.8
Missing	169	25.1	5	10.6	85	25.9	506	38.9	23	37.7	98	36.2
Household composition												
With spouse/children/others	560	83.1	44	93.6	276	84.1	946	72.7	44	72.1	211	77.9
Live alone	64	9.5	2	4.3	22	6.7	254	19.5	10	16.4	42	15.5
Missing	50	7.4	1	2.1	30	9.1	101	7.8	7	11.5	18	6.6
Urbanization												
Urban (>1,500 population/km^2^)	88	13.1	7	14.9	42	12.8	156	12.0	4	6.6	33	12.2
Semi-urban (1,000–1,500 population/km^2^)	258	38.3	12	25.5	113	34.5	448	34.4	20	32.8	78	28.8
Rural (<1,000 population/km^2^)	328	48.7	28	59.6	173	52.7	697	53.6	37	60.7	160	59.0

Body mass index												
<18.5	75	11.1	11	23.4	46	14.0	129	9.9	6	9.8	35	12.9
18.5–24.9	402	59.6	24	51.1	182	55.5	672	51.7	28	45.9	144	53.1
≥25	115	17.1	8	17.0	51	15.5	279	21.4	12	19.7	47	17.3
Missing	82	12.2	4	8.5	49	14.9	221	17.0	15	24.6	45	16.6
Current medical history												
No	409	60.7	19	40.4	170	51.8	644	49.5	31	50.8	138	50.9
Yes	265	39.3	28	59.6	158	48.2	657	50.5	30	49.2	133	49.1
Self-rated health												
Very good/Good	364	54.0	15	31.9	176	53.7	736	56.6	28	45.9	137	50.6
Poor/very poor	283	42.0	31	66.0	144	43.9	511	39.3	32	52.5	122	45.0
Missing	27	4.0	1	2.1	8	2.4	54	4.2	1	1.6	12	4.4
Depressive symptoms (GDS-15)												
No depressive symptoms (GDS-15 <6)	315	46.7	14	29.8	152	46.3	580	44.6	25	41.0	116	42.8
Depressive symptoms (GDS-15 ≥6)	212	31.5	25	53.2	101	30.8	339	26.1	16	26.2	71	26.2
Missing	147	21.8	8	17.0	75	22.9	382	29.4	20	32.8	84	31.0
Intellectual activity												
4 points (full marks)	339	50.3	19	40.4	146	44.5	564	43.4	20	32.8	105	38.7
3 points and under	258	38.3	24	51.1	147	44.8	570	43.8	30	49.2	129	47.6
Missing	77	11.4	4	8.5	35	10.7	167	12.8	11	18.0	37	13.7
Instrumental activities of daily living												
5 points (full marks)	321	47.6	16	34.0	140	42.7	652	50.1	19	31.1	114	42.1
4 points and under	286	42.4	28	59.6	155	47.3	479	36.8	33	54.1	122	45.0
Missing	67	9.9	3	6.4	33	10.1	170	13.1	9	14.8	35	12.9
Frequency of outings												
Higher	518	76.9	30	63.8	243	74.1	949	72.9	37	60.7	185	68.3
Low	99	14.7	15	31.9	60	18.3	240	18.4	16	26.2	61	22.5
Missing	57	8.5	2	4.3	25	7.6	112	8.6	8	13.1	25	9.2

GDS-15, short version of the Geriatric Depression Scale.

**Figure 2.**
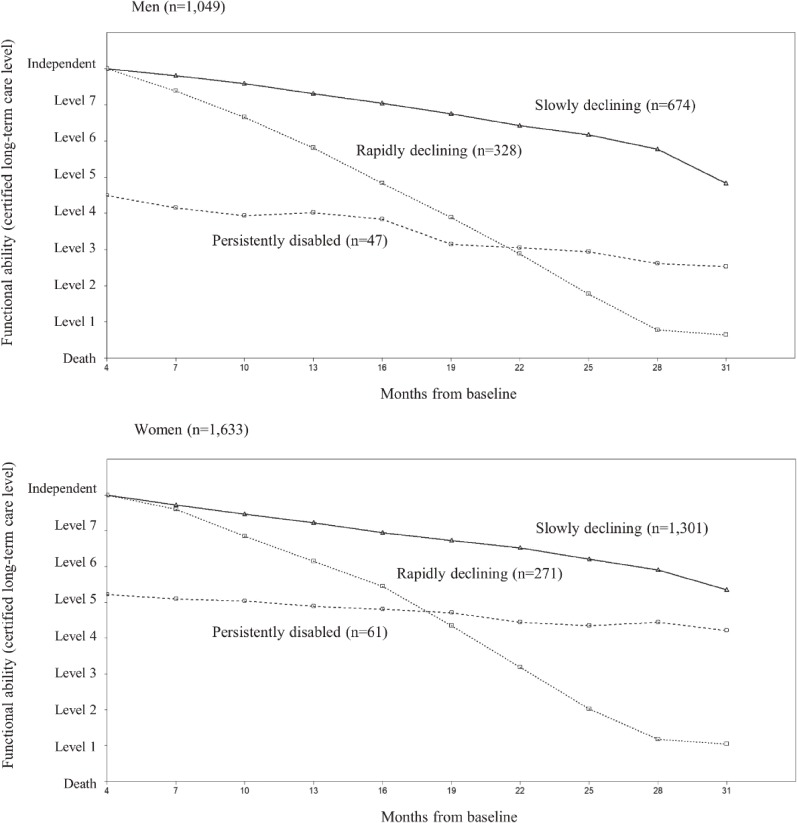
Sample trajectories in functional ability over time by sex. The Y axis shows functional ability (lower care levels indicate worse functional ability). The X axis shows the months from baseline (4 to 31 months). All participants were “independent” in terms of functional ability from 0 (baseline) to 3 months.

We chose a model containing three distinct trajectories of the quadratic type for both men and women. The GMM analysis showed that the BICs for the quadratic trajectories were better than those for the linear trajectories. Furthermore, while the BICs improved as the number of classes increased, we ultimately chose three classes based on the Adj. LMR-LRT (see eTable 1). Figure 2 illustrates the trajectories of certified long-term care levels for men and women, which were categorized as follows: “slowly declining” (674 men [64.3%] and 1,301 women [79.7%]); “persistently disabled” (47 men [4.5%] and 61 women [3.7%]); and “rapidly declining” (328 men [31.3%] and 271 women [16.6%]).

Table 2 shows the results of the multinomial logistic regression analysis by sex. After adjusting for the socio-demographic characteristics (model 1), we found a significant adjusted OR (AOR) for the “persistently disabled” trajectory for men who went outdoors less than once per week (compared with men who went outdoors once per week or more) (AOR 2.68; 95% CI, 1.14–6.28). The results for model 2 (which adjusted for the covariates of model 1 + health status and physiological variables) revealed an attenuated, but still statistically significant, AOR (2.14; 95% CI, 1.03–4.41). However, the AORs for the “rapidly declining” trajectory for both men and women and that for the “persistently disabled” trajectory for women were not statistically significant, although the direction of the associations was positive. We found similar results for the three sensitivity analyses (see eTable 2, eTable 3, and eTable 4).

**Table 2.** Multinomial logistic regression analysis of the “persistently disabled” or “rapidly declining” trajectory compared to the “slowly declining” trajectory by sex

	Men (*n* = 1,049)								Women (*n* = 1,633)							

	Persistently disabled (vs Slowly declining)															
	Model 1^a^				Model 2^b^				Model 1^a^				Model 2^b^			
			
	OR	95% CI		*P*-value	OR	95% CI		*P*-value	OR	95% CI		*P*-value	OR	95% CI		*P*-value
Frequency of outings																
Higher	1.00				1.00				1.00				1.00			
Low	2.68	1.14	6.28	0.023^*^	2.14	1.03	4.41	0.040^*^	1.64	1.02	2.64	0.043^*^	1.20	0.68	2.12	0.524
Missing	0.69	0.10	4.79	0.709	0.74	0.14	3.86	0.725	1.78	1.12	2.83	0.015	1.67	0.61	4.55	0.319

Age																
65–74	1.00				1.00				1.00				1.00			
75–84	1.57	0.69	3.57	0.278	1.50	0.61	3.67	0.379	0.85	0.41	1.75	0.652	0.79	0.35	1.77	0.572
85–	1.09	0.27	4.47	0.900	1.13	0.25	5.08	0.876	0.96	0.61	1.52	0.861	0.78	0.45	1.34	0.361
Education (years)																
≤9	1.00				1.00				1.00				1.00			
≥10	0.35	0.14	0.89	0.027^*^	0.37	0.14	0.96	0.041^*^	0.63	0.38	1.05	0.078	0.67	0.39	1.15	0.150
Missing/others	1.92	0.67	5.50	0.225	1.89	0.68	5.21	0.221	1.00	0.43	2.33	0.991	0.82	0.38	1.74	0.599
Household composition																
With spouse/children/others	1.00				1.00				1.00				1.00			
Live alone	0.67	0.14	3.27	0.620	0.60	0.13	2.84	0.519	1.16	0.57	2.37	0.687	1.25	0.62	2.53	0.529
Missing	0.37	0.05	2.76	0.335	0.41	0.06	2.87	0.372	2.03	0.48	8.57	0.334	2.07	0.43	9.97	0.363
Urbanization																
Urban (>1,500 population/km^2^)	1.00				1.00				1.00				1.00			
Semi-urban (1,000–1,500 population/km^2^)	0.55	0.36	0.84	0.006^*^	0.60	0.39	0.91	0.017^*^	1.71	1.05	2.81	0.032^*^	1.70	1.11	2.61	0.016^*^
Rural (<1,000 population/km^2^)	0.93	0.56	1.54	0.786	0.99	0.56	1.75	0.968	1.98	1.81	2.16	<0.001	2.02	1.77	2.32	<0.001

Body mass index																
<18.5					2.33	1.24	4.40	0.009^*^					1.06	0.40	2.84	0.900
18.5–24.9					1.00								1.00			
≥25					1.26	0.41	3.87	0.685					1.08	0.56	2.10	0.822
Missing					1.10	0.34	3.60	0.874					1.66	0.76	3.63	0.203
Current medical history																
No					1.00								1.00			
Yes					1.93	0.90	4.14	0.092					0.75	0.48	1.16	0.198
Instrumental activities of daily living																
5 points (full marks)					1.00								1.00			
4 points and under					1.15	0.75	1.76	0.523					2.27	1.42	3.64	0.001^*^
Missing					0.95	0.20	4.64	0.952					1.04	0.34	3.17	0.944



	Rapidly declining (vs Slowly declining)															
	Model 1^a^				Model 2^b^				Model 1^a^				Model 2^b^			
			
	OR	95% CI		*P*-value	OR	95% CI		*P*-value	OR	95% CI		*P*-value	OR	95% CI		*P*-value
Frequency of outings																
Higher	1.00				1.00				1.00				1.00			
Low	1.16	0.88	1.53	0.291	1.11	0.82	1.50	0.498	1.29	0.96	1.73	0.086	1.12	0.87	1.44	0.388
Missing	0.88	0.48	1.61	0.675	0.91	0.53	1.55	0.723	1.21	0.68	2.18	0.519	1.10	0.50	2.42	0.805

Age																
65–74	1.00				1.00				1.00				1.00			
75–84	0.64	0.51	0.80	<0.001^*^	0.61	0.48	0.77	<0.001^*^	0.66	0.50	0.87	0.004^*^	0.63	0.50	0.79	<0.001^*^
85–	0.93	0.55	1.60	0.799	0.87	0.50	1.51	0.613	0.57	0.33	0.98	0.042^*^	0.50	0.33	0.76	0.001^*^
Education (years)																
≤9	1.00				1.00				1.00				1.00			
≥10	0.84	0.68	1.03	0.087	0.87	0.68	1.10	0.251	0.75	0.58	0.97	0.026^*^	0.74	0.58	0.95	0.017^*^
Missing/others	1.38	0.70	2.73	0.350	1.31	0.66	2.58	0.437	1.02	0.66	1.57	0.923	0.98	0.55	1.73	0.938
Household composition																
With spouse/children/others	1.00				1.00				1.00				1.00			
Live alone	0.59	0.35	1.00	0.051	0.58	0.32	1.06	0.075	0.71	0.53	0.94	0.018^*^	0.74	0.55	0.99	0.042^*^
Missing	1.22	0.85	1.73	0.279	1.24	0.83	1.85	0.296	0.86	0.51	1.46	0.585	0.82	0.44	1.53	0.531
Urbanization																
Urban (>1,500 population/km^2^)	1.00				1.00				1.00				1.00			
Semi-urban (1,000–1,500 population/km^2^)	0.93	0.66	1.30	0.679	0.95	0.69	1.32	0.768	0.85	0.69	1.04	0.111	0.82	0.66	1.02	0.069
Rural (<1,000 population/km^2^)	1.13	0.79	1.60	0.504	1.17	0.84	1.63	0.343	1.10	0.90	1.35	0.361	1.10	0.90	1.35	0.348

Body mass index																
<18.5					1.41	0.89	2.24	0.144					1.23	0.80	1.89	0.355
18.5–24.9					1.00								1.00			
≥25					0.96	0.70	1.33	0.818					0.75	0.59	0.94	0.013^*^
Missing					1.23	0.80	1.89	0.345					1.01	0.68	1.51	0.952
Current medical history																
No					1.00								1.00			
Yes					1.55	1.20	2.01	0.001^*^					0.89	0.64	1.24	0.496
Instrumental activities of daily living																
5 points (full marks)					1.00								1.00			
4 points and under					1.09	0.70	1.71	0.694					1.44	0.95	2.19	0.086
Missing					1.14	0.50	2.62	0.752					1.02	0.58	1.81	0.935

CI, confidence interval; GDS-15, short version of the Geriatric Depression Scale; OR, odds ratio.

Only covariates that are significantly related to at least one of the trajectories are listed.

^a^Adjusted for age, marital status, education years, equivalent annual household income, household composition, and urbanization.

^b^Adjusted for the covariates in Model 1 + body mass index, current medical history, self-rated health, depression (GDS-15 ≥6), intellectual activities, and instrumental activities of daily living.

^*^P < 0.05.

## DISCUSSION

Our models suggested three distinct trajectories of functional ability for both men and women. Moreover, men with a low frequency of outings at baseline had 2.14 times greater odds of belonging to the “persistently disabled” trajectory, rather than the “slowly declining” trajectory, compared to men with a higher frequency of outings after adjusting for potential confounders.

The identified trajectories were consistent with those identified in previous studies.^3^^–^^5^ However, we expanded on past evidence by examining the trajectories from the first occurrence of functional disability, along with how social activity predicts these trajectories. Yu et al found that leisure activities were related to a “functional maintenance” trajectory (which is a desirable pattern)^7^; however, they included people with functional disabilities at baseline. In this study, we limited participants to those who were independent at baseline to decrease the possibility of reverse causation.

Several plausible mechanisms might explain the link between frequency of outings and the trajectories of functional decline among older men. First, a low frequency of outings may lead to limited social relationships, which in turn could directly lead to the deterioration of functional ability. Outing frequency is directly related to the size of one’s social network,^14^ which promotes healthy behaviors by increasing opportunities to access health services and information.^21^^,^^22^ Furthermore, social relationships might help prevent rapid deterioration in functional ability via social support, which can indirectly affect health by improving mental health.^22^ In an intervention study examining how community salons affect older people, the risk of being certified for long-term care was halved. The suggested mechanism of this was that participating in these salons expanded participants’ social network, thus enabling them to obtain greater social support.^23^

Another possibility is that outing frequency reflects the level of physical activity, such as walking. The frequency of outings is significantly associated with physical activity level,^24^ and exercise (including walking) is effective for improving cognitive function.^25^^,^^26^ Thus, remaining in the house might lead to a decrease in the body’s functional or cognitive reserves, leading to a continual decline in physical or cognitive resilience until they require long-term care.

Alternatively, however, it may also be possible that people who belonged to the “persistently disabled” group already had a tendency to stay in their houses at the time of baseline because their functions were somewhat weak. Physical or cognitive functions might not be the same at the baseline for all study participants, even though none of them needed nursing care. Therefore, the study results need cautious interpretation and further research is required to confirm that the observed associations are causal.

An association between frequency of outings and the “persistently disabled” trajectory was found in this study, but only in men. This gender difference is consistent with previous findings, which suggest that a low frequency of outings is a greater risk for the onset of functional decline in men than in women.^27^ One of the potential reasons for this may be that men and women tend to build their social relations in different places. When the study participants were still in the workforce, their social network mainly comprised work colleagues for men and individuals in the community for women (given that many of them were homemakers in Japan).^28^ As a result, women who are aged over 65 years old—even those who only go out less than once per week—might receive more social support from people in their communities.^29^ Furthermore, compared with men who remain in the house, women who do so might still have higher levels of physical activity because of their greater participation in housekeeping.^30^

A strength of this study was its use of a prospective cohort design with large panel data at 10 time points. Additionally, we used an objective indicator of functional ability as an outcome measurement, and controlled for various confounders, including socio-demographics and physiological variables. This study also has several limitations. First, the data on long-term care levels were obtained only for people who applied for the LTCI. Individuals who did not use public LTCI for any reason, such as being able to afford private nursing care, would be considered independent in our analysis. However, as a rule, all people living in Japan over 40 years old are insured by the LTCI; therefore, we assume that few people in need of LTCI do not apply for it. Second, reverse causation remains possible, as we did not adjust for some confounders relating to the preliminary stage of functional decline. To improve our inferences of causality, we restricted study participants to those who were independent at baseline, excluded persons who were certified for long-term care within 3 months after the baseline, and adjusted for the major diseases related to long-term care level and ADLs at baseline. Third, the magnitudes of the gap between each functional ability levels are not even across the levels evaluated using national LTCI system and mortality information. Moreover, death is conceptually different from functional ability. The potential solution may be to exclude cases of death. However, we believe that it is important to understand the patterns of declining functional ability among all older people who can be the targets of long-term care prevention measures in the community. Thus, we have conceptualized death as being at the end of the continuum of functional disability. Finally, the database contained no information on those who were certified as “non-applicable (independent)” after initially being certified as needing long-term care. This indicates that individuals whose long-term care level ultimately reached independence were considered to have maintained the care level that they previously had. However, in a 5-year period, such cases account for less than 1% of all certified persons requiring long-term care.^31^

In conclusion, we identified three distinct trajectories of functional abilities among community-dwelling older people during a 31-month observation period. Furthermore, a low frequency of outings was associated with a future pattern of maintaining moderate functional decline among older men, but not with a future rapid decline. The study results need to be cautiously interpreted due to the possible reverse causation that weaker functions at baseline caused fewer outings. If the observed association was causal, interventions to promote outings for older people, such as increasing opportunities to access community events and ease of access to restaurants and retail stores,^32^ might be effective in slowing the decline of functional ability among men with better initial care levels. It also would be necessary to consider the sex-specific factors related to a low frequency of outings.

## APPENDIX A. SUPPLEMENTARY DATA

eTable 1. Fit statistics for growth mixture model (GMM)

eTable 2. Multinomial logistic regression analysis among participants by sex after excluding those who died in the first 6 months from baseline

eTable 3. Multinomial logistic regression analysis among participants by sex, excluding those aged 85 years or more

eTable 4. Multinomial logistic regression analysis after adjusting for long-term care levels at the initial certification.
