# Supplementary material for: Exploring 2.5-Year Trajectories of Functional Decline in Older Adults by Applying a Growth Mixture Model and Frequency of Outings as a Predictor: A 2010–2013 JAGES Longitudinal Study
Source: J Epidemiol. 2019 Feb 5;29(2):65–72. doi: 10.2188/jea.JE20170230 (PMC6336721; doi:10.2188/jea.JE20170230)
Supplement: Supplementary file 1 [file je-29-065-s001.pdf]

eTable 1. Fit statistics for growth mixture model (GMM)

	Men (n = 1,049)				Women (n = 1,633)		
	2 classes	3 classes	4 classes		2 classes	3 classes	4 classes
Quadratic				Quadratic			
BIC	29259	28879	28786	BIC	39141	38502	38250
Adj.LMR-LRT (p)	35318 (<0.001)	1053643 (<0.001)	139 (0.39)	Adj.LMR-LRT (p)	51039 (<0.001)	983 (<0.001)	300 (0.14)
Group size (%)				Group size (%)			
Class 1	47 (4.5%)	47 (4.5%)	47 (4.5%)	Class 1	61 (3.7%)	61 (3.7%)	61 (3.7%)
Class 2	1002 (95.5%)	328 (31.3%)	277 (26.4%)	Class 2	1572 (96.3%)	271 (16.6%)	187 (11.5%)
Class 3		674 (64.3%)	212 (20.2%)	Class 3		1301 (79.7%)	562 (34.4%)
Class 4			513 (48.9%)	Class 4			823 (50.4%)
Linear				Linear			
BIC	31694	31572	31481	BIC	42563	42415	42336
Adj.LMR-LRT (p)	7286 (<0.001)	154 (0.19)	124 (0.019)	Adj.LMR-LRT (p)	10897 (<0.001)	346 (0.047)	114 (0.002)
Group size(%)				Group size(%)			
Class 1	47 (4.5%)	20 (1.9%)	20 (1.9%)	Class 1	61 (3.7%)	10 (0.6%)	10 (0.6%)
Class 2	1002 (95.5%)	27 (2.6%)	27 (2.6%)	Class 2	1572 (96.3%)	51 (3.1%)	51 (3.1%)
Class 3		1002 (95.5%)	138 (13.2%)	Class 3		1572 (96.3%)	148 (9.1%)
Class 4			864 (82.4%)	Class 4			1424 (87.2%)

Adj. LMR-LRT, adjusted Lo-Mendell-Rubin likelihood ratio test; BIC, Bayesian information criterion.

*In cubic models, the BIC and Adj.MR-LRT were not replicated because of an ill-conditioned Fisher information matrix, which suggested that we need to change the model.

eTable 2. Multinomial logistic regression analysis among participants by sex after excluding those who died in the first 6 months from baseline

Men (n = 1,042)

	Persistently disabled (vs. Slowly declining)							
	Model 1				Model 2			
	OR	95% CI		P-value	OR	95% CI		P-value
Frequency of going outdoors								
Higher	1.00				1.00			
Low	2.55	1.05	6.20	0.039 *	2.07	0.96	4.43	0.062
Missing	0.36	0.05	2.51	0.303	0.44	0.07	2.87	0.393
	Rapidly declining (vs. Slowly declining)							
	Model 1				Model 2			
	OR	95% CI		P-value	OR	95% CI		P-value
Frequency of going outdoors								
Higher	1.00				1.00			
Low	1.16	0.87	1.53	0.309	1.11	0.81	1.51	0.526
Missing	0.89	0.48	1.64	0.699	0.91	0.53	1.57	0.746

Women (n = 1,631)

	Persistently disabled (vs. Slowly declining)							
	Model 1				Model 2			
	OR	95% CI		P-value	OR	95% CI		P-value
Frequency of going outdoors								
Higher	1.00				1.00			
Low	1.44	0.89	2.32	0.138	1.05	0.6	1.83	0.866
Missing	1.77	1.12	2.78	0.014 *	1.64	0.59	4.54	0.343
	Rapidly declining (vs. Slowly declining)							
	Model 1				Model 2			
	OR	95% CI		P-value	OR	95% CI		P-value
Frequency of going outdoors								
Higher	1.00				1.00			
Low	1.30	0.97	1.73	0.082	1.12	0.87	1.44	0.370
Missing	1.21	0.68	2.18	0.518	1.1	0.5	2.42	0.804

CI, confidence interval; GDS-15, short version of the Geriatric Depression Scale;
OR, odds ratio.
Model 1: adjusted for age, marital status, education years, equivalent annual household income, household composition, and urbanization
Model 2: adjusted for the covariates in model 1 + body mass index, current medical history, self-rated health, depression (GDS-15 ≥6), intellectual activities, and instrumental activities of daily living
*p<0.05

eTable 3. Multinomial logistic regression analysis among participants by sex, excluding those aged 85 years or more

Men (n = 830)

	Persistently disabled (vs. Slowly declining)							
	Model 1				Model 2			
	OR	95% CI		P-value	OR	95% CI		P-value
Frequency of going outdoors								
Higher	1.00				1.00			
Low	4.15	2.04	8.42	<0.001 *	3.22	1.67	6.21	<0.001 *
Missing	0.89	0.11	7.17	0.915	0.91	0.15	5.52	0.919

	Rapidly declining (vs. Slowly declining)							
	Model 1				Model 2			
	OR	95% CI		P-value	OR	95% CI		P-value
Frequency of going outdoors								
Higher	1.00				1.00			
Low	1.30	0.84	2.03	0.243	1.30	0.79	2.12	0.308
Missing	0.89	0.45	1.76	0.733	0.98	0.55	1.75	0.949

Women (n = 1,228)

	Persistently disabled (vs. Slowly declining)							
	Model 1				Model 2			
	OR	95% CI		P-value	OR	95% CI		P-value
Frequency of going outdoors								
Higher	1.00				1.00			
Low	1.64	1.10	2.44	0.015 *	1.15	0.65	2.03	0.630
Missing	2.13	1.19	3.81	0.011 *	1.54	0.60	3.92	0.366

	Rapidly declining (vs. Slowly declining)							
	Model 1				Model 2			
	OR	95% CI		P-value	OR	95% CI		P-value
Frequency of going outdoors								
Higher	1.00				1.00			
Low	1.16	0.80	1.69	0.434	1.02	0.68	1.53	0.914
Missing	1.40	0.69	2.84	0.345	1.16	0.41	3.30	0.783

CI, confidence interval; GDS-15, short version of the Geriatric Depression Scale;
OR, odds ratio.
Model 1: adjusted for age, marital status, education years, equivalent annual household income, household composition, and urbanization
Model 2: adjusted for the covariates in model 1 + body mass index, current medical history, self-rated health, depression (GDS-15 ≥6), intellectual activities, and instrumental activities of daily living
*p<0.05

eTable 4. Multinomial logistic regression analysis after adjusting for long-term care levels at the initial certification.

Men (n = 1,049)

	Persistently disabled (vs. Slowly declining)							
	Model 1				Model 2			
	OR	95% CI		P-value	OR	95% CI		P-value
Frequency of going outdoors								
Higher	1.00				1.00			
Low	2.62	1.11	6.17	0.027 *	2.14	1.03	4.41	0.040 *
Missing	0.68	0.10	4.53	0.689	0.75	0.15	3.78	0.729
	**Rapidly declining (vs. Slowly declining)**							
	Model 1				Model 2			
	OR	95% CI		P-value	OR	95% CI		P-value
Frequency of going outdoors								
Higher	1.00				1.00			
Low	1.11	0.82	1.52	0.487	1.13	0.82	1.58	0.451
Missing	0.84	0.48	1.47	0.541	0.96	0.57	1.64	0.889

Women (n = 1,633)

	Persistently disabled (vs. Slowly declining)							
	Model 1				Model 2			
	OR	95% CI		P-value	OR	95% CI		P-value
Frequency of going outdoors								
Higher	1.00				1.00			
Low	1.63	1.03	2.59	0.036 *	1.22	0.71	2.10	0.474
Missing	1.78	1.12	2.82	0.015	1.69	0.62	4.64	0.308
	**Rapidly declining (vs. Slowly declining)**							
	Model 1				Model 2			
	OR	95% CI		P-value	OR	95% CI		P-value
Frequency of going outdoors								
Higher	1.00				1.00			
Low	1.09	0.77	1.54	0.614	1.02	0.77	1.37	0.870
Missing	1.05	0.54	2.05	0.880	1.00	0.45	2.24	0.998

CI, confidence interval; GDS-15, short version of the Geriatric Depression Scale;
OR, odds ratio.
Model 1: adjusted for age, marital status, education years, equivalent annual household income, household composition, urbanization, and the initial long-term care level
Model 2: adjusted for the covariates in model 1 + body mass index, current medical history, self-rated health, depression (GDS-15 ≥6), intellectual activities, and instrumental activities of daily living
*p<0.05
